# Spicy food consumption is associated with adiposity measures among half a million Chinese people: the China Kadoorie Biobank study

**DOI:** 10.1186/1471-2458-14-1293

**Published:** 2014-12-17

**Authors:** Dianjianyi Sun, Jun Lv, Wei Chen, Shengxu Li, Yu Guo, Zheng Bian, Canqing Yu, Huiyan Zhou, Yunlong Tan, Junshi Chen, Zhengming Chen, Liming Li, On behalf of the China Kadoorie Biobank collaborative group

**Affiliations:** Department of Epidemiology and Biostatistics, School of Public Health, Peking University Health Science Center, 38 Xueyuan Road, Beijing, 100191 China; Department of Epidemiology, School of Public Health and Tropical Medicine, Tulane University, New Orleans, LA USA; Chinese Academy of Medical Sciences, Beijing, China; China National Center for Food Safety Risk Assessment, Beijing, China; Clinical Trial Service Unit & Epidemiological Studies Unit (CTSU), Nuffield Department of Population Health, University of Oxford, UK, Old Road Campus, Oxford, OX3 7LF UK

**Keywords:** Spicy food intake, Body mass index, Central obesity

## Abstract

**Background:**

Few animal experiments and volunteer-based intervention studies have showed a controversial effect of spicy foods on weight management; however, information is scant on the association between spicy food intake and obesity. This study aims to examine the impact of spicy food on quantitative adiposity measures in the Chinese population; a population with a low prevalence of general obesity, but a high prevalence of central obesity.

**Methods:**

A total of 434,556 adults (255,094 females), aged 30–79 years, were included from the China Kadoorie Biobank (CKB) study. Information on spicy food intake was obtained using a questionnaire survey. Body mass index (BMI), percentage body fat (BF%), waist circumference (WC), and WC/height ratio (WHtR) were analyzed as continuous variables.

**Results:**

The prevalence of daily spicy food eating was 30.4% in males and 30.0% in females, with dramatically geographic diversity (ranging from 99.4% in Hunan to 2.7% in Zhejiang). The covariates-adjusted BMI, BF%, WC, and WHtR significantly increased with increasing frequency, strength, and duration of spicy food eating regardless of gender (p < 0.001). Among regular spicy food consumers, strength of spicy food eating showed significant and positive association with all adiposity measures in both genders (except for BF% in males). Compared with non-consumers, daily spicy food eating was significantly associated with an increase of 0.44 and 0.51 of BMI (kg/m^2^), 0.79 and 1.01 of BF%, 1.4 and 1.0 of WC (cm), and 0.008 and 0.006 of WHtR in males and females, respectively. In stratified analyses of 18 consecutive BMI subgroups, a significantly increasing trend in the effect of daily spicy food eating on WC and WHtR with increasing BMI was noted in males; whereas a decreasing trend was seen in females.

**Conclusions:**

The data indicate that spicy food intake is a risk factor for obesity in Chinese adult population, especially for central obesity in males. Further studies are needed to elucidate the mechanisms underlying this association.

## Background

Spiciness or pungency, regarded as one of the basic tastes in ancient India and China [1], is actually a sensation of heat caused by the activation of pain receptors in the tongue of mammals [2]. Although some previous studies have shown beneficial effects of spicy food consumption on chronic diseases and other conditions [2–5], inconsistent results of the association between spicy food intake and obesity have been reported in animal and human intervention studies regarding different types like supplements, drugs or food and the mechanisms underlying the energy expenditure and appetite changes [2, 6–8]. Compared with Western societies, Asian populations have a relatively lower body mass index (BMI) but are predisposed to central or abdominal obesity [9, 10]. To date, the association of spicy foods eating with obesity and central obesity has never been examined in Asian populations.

As obesity has reached an epidemic level worldwide [11], it is of great public interest to explore whether spicy foods as attractive supplements may contribute to body weight management [12]. However, previous findings in this regard are mainly from animal experiments [13–16] and intervention studies with a small number of volunteers [6–8, 17–19]; no observational studies have focused on this aspect. The present study aims to examine the impact of spicy food eating on BMI, percentage body fat (BF%), waist circumference (WC), and WC-to-height ratio (WHtR) in 0.5 million adults enrolled in the China Kadoorie Biobank (CKB) study.

## Methods

### Study design and participants

The CKB Study is a prospective study of chronic disease in China. Details of the CKB study design and characteristics of the study participants have been described elsewhere [20, 21]. Briefly, 512,891 participants without major disabilities living in administrative units (rural villages or urban residential committees), aged 30–79 years (mean age: 51.5 years), were recruited in the baseline survey between 2004 and 2008 from five urban and five rural areas in China. Selection of the survey sites was based on local patterns of disease and exposure to certain risk factors, population stability, quality of death and disease registries, local commitment, and capacity.

The current analysis included participants who had a weight between 30 kg and 160 kg, a height of 145–200 cm (males) and 140–200 cm (females), a BMI between 18.5 and 45.0, and a waist circumference between 50 cm and 150 cm. A number of 25,364 participants were excluded in accord with the anthropometric inclusion criteria. People who were very underweight, extremely obese, or were at the extremes of the height distribution may have underlying metabolic or growth disorders. Additionally, a total number of 56,144 participants with certain self-reported disease history were excluded, including peptic ulcer, cirrhosis, chronic hepatitis, gallstone, gallbladder disease, or any type of cancer, which may bias the association between body weight and spicy food. A total of 78,335 participants were excluded based on the above selection criteria, and 434,556 adults (179,462 males and 255,094 females) formed the sample for the current analysis.

Ethical approval for the CKB study was obtained from the Ethical Review Committee of the Chinese Center for Disease Control and Prevention (Beijing, China) and the Oxford Tropical Research Ethics Committee, University of Oxford (UK). In addition, approvals were obtained from the institutional research boards at the local Center for Disease Control and Prevention in each of the ten survey sites. Finally, written informed consent was obtained from all participants.

### Questionnaire survey

In the baseline survey, trained interviewers administered a standardized questionnaire using a laptop-based direct data-entry system, with built-in functions to avoid logical errors and missing items. The questionnaire included detailed questions on socio-demographic status, medical history and health status, spicy food eating habits, tobacco and alcohol consumption, physical activity, and other lifestyle behaviors.

For assessment of spicy food intake, participants were asked how often they had eaten hot spicy food during the past month: 1) Never and only occasionally; 2) 1–2 days per week; 3) 3–5 days per week; 4) daily or almost every day. Among those who ate spicy food more than once a week, participants were asked at what age they started to eat spicy food, and the strength of spicy food eating they usually preferred. The strength of spicy food eating was categorized as weak, moderate and strong. Further, years of eating spicy food-to-current age ratio is used as a measure of a life-long burden of spicy food consumption.

For assessment of alcohol consumption and smoking, participants were asked how often they had drunk alcohol during the previous 12 months and smoked tobacco at the time of the survey, and those who had not drunk or smoked weekly were asked if there was a period of at least a year prior to that when they had drunk some alcohol at least once a week or had smoked some tobacco on most days or daily. Participants were classified into four main drinking or smoking categories: 1) nondrinkers (or nonsmokers); 2) ex-drinkers (or ex-smokers); 3) occasional drinkers (or occasional smokers); and 4) weekly drinkers (or weekly smokers).

Total physical activity was converted into metabolic equivalent hours per day (MET-hours/day) spent on work, transportation, housework, and non-sedentary recreation as described in our previous study [22].

### Anthropometric measurements

Standing height and body weight were measured in light indoor clothing without shoes to the nearest 0.1 cm and 0.1 kg, respectively. BMI was calculated as weight in kilograms divided by the square of height in meters. BF% was measured by using a bioelectrical impedance device (TANITA-TBF-300GS; Tanita Corp). WC was measured midway between the iliac crest and the lower rib margin at the end of normal expiration using a plastic flexible tape to the nearest 0.1 cm. All measurements were made by trained staff using a standard protocol and instruments. WHtR was calculated as WC divided by height; WC and WHtR were used as quantitative measures of central obesity; [23].

### Statistical analyses

Statistical analyses were performed using Stata (version 13.1, 2013, StataCorp LP., College Station, TX). All analyses were performed separately by gender groups. The descriptive data were presented as covariates-adjusted means and standard error (SE) for continuous variables derived from multivariable linear regression, and adjusted percentages and SE for categorical variables estimated using multinomial logistic regression models. General linear models were used to test differences and trends in continuous study variables across subgroups, adjusting for age and survey sites. Chi-square test was used to test the differences in categorical study variables across subgroups.

Multivariable linear regression analyses were performed (dependent variables = BMI, BF%, WC, or WHtR; predictors = frequency of spicy food eating, strength of spicy food eating, and ratio of years of eating spicy food to age), adjusting for age, survey sites (included in the regression models as nine dummy variables using Qingdao as a reference), education (categorized as Illiterate and Elementary, middle school, high school and above), total physical activity (MET-hours/day), and alcohol and tobacco use.

A fixed-effect or random-effect model was used for meta-analysis of the overall association of daily spicy food eating with BMI, BF%, WC, and WHtR in individual survey sites by gender groups. Heterogeneity in the site-specific association parameters was evaluated by χ^2^ and I^2^. The hypothesis of homogeneity between subgroups was rejected if p-value was <0.1 (Cochran Q tests) [24]. Then the random-effect model was adopted, and the cause of heterogeneity was investigated.

In order to assess the association strengths of spicy food intake with BF%, WC, and WHtR at different BMI levels, males and females were stratified into 18 subgroups according to levels of BMI with equal numbers of subjects in each subgroup. Multivariable linear regression analyses were performed within each subgroup, adjusting for covariates as mentioned above. Standardized regression coefficients of daily spicy food eating associated with BF%, WC, and WHtR in the 18 BMI subgroups were plotted with means of BMI of the 18 subgroups in males vs females. Linear regression models were used to assess the significance of trends of the effect of spicy food on BF%, WC, and WHtR across these 18 BMI subgroups; interaction regression models were used to test the difference in slopes of the regression lines between gender groups.

## Results

Table 1 summarizes the characteristics of the study cohort by gender and frequency of spicy food eating with adjustment for age and survey sites. The daily spicy food consumers accounted for 30.4% in males and 30.0% in females, and most of them were from rural areas (81.8% in males and 81.1% in females). Except for non-spicy food eaters, the proportion of illiterate and elementary education level increased with increasing frequency of spicy food eating in both genders. Moreover, the higher the frequency of spicy food eating, the higher the physical activity level, as well as the prevalence of current regular alcohol drinkers and smokers regardless of gender. Among those who ate spicy food regularly (participants who ate spicy food more than once a week), the more frequently who ate spicy food, and the stronger the intensity of spicy flavor who preferred, and the longer the duration of eating spicy food.

**Table 1:** **Characteristics of study variables by gender and frequency of spicy food eating**

	Male (n = 179,462)				Female (n = 255,094)			
	Never (n = 100,604)	1-2 d/wk (n = 12,813)	3-5 d/wk (n = 11,408)	Daily (n = 54,637)	Never (n = 149,106)	1-2 d/wk (n = 15,422)	3-5 d/wk (n = 13,999)	Daily (n = 76,567)
Age (year)	53.0 (0.03)	49.3 (0.09)	49.2 (0.10)	51.1 (0.05)	51.8 (0.03)	48.0 (0.08)	48.1 (0.09)	48.6 (0.04)
Study area (%)^a^								
Rural	47.7 (0.2)	40.1 (0.4)	39.0 (0.5)	81.8 (0.2)	45.2 (0.1)	40.5 (0.4)	40.9 (0.4)	81.1 (0.1)
Urban	52.3 (0.2)	59.9 (0.4)	61.0 (0.5)	18.2 (0.2)	54.8 (0.1)	59.5 (0.4)	59.1 (0.4)	18.9 (0.1)
Education level (%)^b^								
Illiterate and Elementary	42.7 (0.2)	39.9 (0.4)	39.5 (0.4)	40.5 (0.2)	57.4 (0.1)	53.3 (0.3)	52.5 (0.4)	53.7 (0.2)
Middle school	32.6 (0.2)	32.5 (0.4)	33.6 (0.4)	34.0 (0.3)	25.1 (0.1)	26.2 (0.3)	27.3 (0.4)	27.8 (0.2)
High school and above	24.8 (0.1)	27.5 (0.3)	26.9 (0.4)	25.5 (0.2)	17.5 (0.1)	20.5 (0.3)	20.2 (0.3)	18.5 (0.2)
Physical activity (MET-hours/day)^b^	22.4 (0.05)	22.1 (0.12)	22.4 (0.13)	22.5 (0.08)	20.3 (0.03)	20.7 (0.09)	21.1 (0.09)	20.9 (0.05)
Alcohol use (%)^b^								
Nondrinker	24.2 (0.17)	17.6 (0.35)	15.2 (0.35)	13.5 (0.15)	66.7 (0.12)	58.6 (0.33)	56.9 (0.35)	56.6 (0.20)
Ex-regular drinker	8.4 (0.11)	7.7 (0.26)	7.3 (0.27)	6.8 (0.13)	0.7 (0.03)	0.9 (0.08)	1.0 (0.09)	0.9 (0.03)
Occasional drinker	40.0 (0.18)	37.4 (0.42)	35.9 (0.44)	32.1 (0.25)	31.4 (0.12)	38.1 (0.32)	38.9 (0.34)	38.8 (0.19)
Regular drinker	27.4 (0.14)	37.3 (0.40)	41.6 (0.44)	47.6 (0.28)	1.2 (0.03)	2.4 (0.12)	3.1 (0.14)	3.7 (0.10)
Tobacco use (%)^b^								
Nonsmoker	17.8 (0.15)	13.5 (0.31)	12.2 (0.31)	10.1 (0.16)	96.4 (0.05)	95.1 (0.16)	94.3 (0.18)	94.3 (0.09)
Ex-regular smoker	13.3 (0.11)	12.4 (0.30)	12.2 (0.32)	11.8 (0.20)	0.7 (0.02)	0.7 (0.07)	0.9 (0.08)	0.9 (0.04)
Occasional smoker	13.1 (0.13)	11.9 (0.30)	11.1 (0.30)	8.8 (0.15)	1.3 (0.04)	2.2 (0.12)	2.3 (0.12)	2.1 (0.06)
Regular smoker	55.8 (0.18)	62.1 (0.44)	64.5 (0.45)	69.3 (0.26)	1.6 (0.04)	1.9 (0.11)	2.4 (0.12)	2.8 (0.07)
Strength of spicy food eating (%)^b^								
Weak								
Moderate	NA	48.2 (0.4)	35.8 (0.4)	17.9 (0.2)	NA	52.9 (0.4)	40.4 (0.3)	19.5 (0.2)
Strong	NA	34.8 (0.5)	35.7 (0.5)	38.8 (0.2)	NA	34.2 (0.5)	34.4 (0.4)	38.0 (0.2)
Duration of spicy food eating (%)^b^	NA	17.1 (0.5)	28.5 (0.4)	43.3 (0.2)	NA	12.9 (0.5)	25.1 (0.4)	42.4 (0.2)
Age started to eat spicy food regularly (year)	NA	18.4 (0.08)	17.8 (0.08)	15.5 (0.04)	NA	18.0 (0.07)	16.8 (0.07)	14.0 (0.03)
Number of years of eating spicy food^#^	NA	32.1 (0.08)	32.8 (0.08)	35.0 (0.04)	NA	30.5 (0.07)	31.7 (0.07)	34.5 (0.03)
Years of eating spicy food-to-age ratio (%)^#^	NA	57.2 (0.2)	58.5 (0.2)	63.4 (0.1)	NA	56.9 (0.1)	59.4 (0.1)	65.5 (0.1)

Data are presented as adjusted mean (SE) or percentage (SE). Adjusted means and proportions were calculated by using multiple linear regression and multinomial logistic regression models, respectively. All P-heterogeneity and P-trend across subgroups are <0.001.

^a^adjusted for age; ^b^, adjusted for age and survey sites; ^#^sample size are 788,853 in males and 105,982 in females.

Table 2 gives covariates-adjusted means of adiposity measures (BMI, BF%, WC, and WHtR) according to frequency, strength, and years of eating spicy food-to-age ratio in males and females. Females had higher BMI, BF%, and WHtR but lower WC than males. Of note, higher BMI, BF%, WC, and WHtR were associated with higher frequency, stronger flavor, or longer duration of spicy food eating in both genders (p for trend < 0.001).

**Table 2:** **Characteristics of adiposity variables by gender and spicy food eating groups**

	BMI		BF%		WC		WHtR	
	Males	Females	Males	Females	Males	Females	Males	Females
**Frequency of spicy food eating**								
**n**	179,462	255,094	179,365	254,997	179,462	255,094	179,462	255,094
Never	23.6 (0.01)	23.9 (0.01)	22.2 (0.02)	32.3 (0.02)	82.2 (0.03)	79.3 (0.03)	0.498 (0.0002)	0.514 (0.0002)
1-2 d/wk	23.8 (0.03)	24.1 (0.03)	22.6 (0.05)	32.6 (0.05)	83.1 (0.08)	79.8 (0.07)	0.502 (0.0005)	0.517 (0.0004)
3-5 d/wk	23.9 (0.03)	24.2 (0.03)	22.6 (0.05)	32.9 (0.06)	83.2 (0.08)	79.9 (0.07)	0.503 (0.0005)	0.518 (0.0005)
Daily	24.0 (0.02)	24.4 (0.02)	22.9 (0.03)	33.3 (0.03)	83.5 (0.05)	80.4 (0.04)	0.505 (0.0003)	0.521 (0.0003)
**Strength of spicy food eating**								
**n**	78,858	105,988	78,808	105,931	78,858	105,988	78,858	105,988
Weak	23.4 (0.02)	23.5 (0.02)	22.0 (0.04)	31.8 (0.04)	81.8 (0.07)	78.5 (0.05)	0.497 (0.0004)	0.511 (0.0003)
Moderate	23.5 (0.02)	23.8 (0.02)	22.2 (0.04)	32.1 (0.03)	82.2 (0.05)	79.1 (0.05)	0.500 (0.0003)	0.515 (0.0003)
Strong	23.5 (0.02)	24.0 (0.02)	22.3 (0.04)	32.6 (0.04)	82.5 (0.06)	79.7 (0.05)	0.502 (0.0004)	0.518 (0.0003)
**Years of eating spicy food-to-age ratio**								
**n**	78,853	105,982	78,804	105,925	78,853	105,982	78,853	105,982
<50%	23.4 (0.03)	23.6 (0.03)	22.0 (0.06)	31.7 (0.06)	81.9 (0.09)	78.7 (0.08)	0.498 (0.0005)	0.512 (0.0005)
> = 50%	23.5 (0.02)	23.8 (0.02)	22.2 (0.04)	32.2 (0.04)	82.4 (0.06)	79.2 (0.05)	0.500 (0.0004)	0.515 (0.0003)
> = 80%	23.5 (0.02)	23.9 (0.02)	22.3 (0.04)	32.4 (0.04)	82.3 (0.06)	79.3 (0.05)	0.500 (0.0004)	0.517 (0.0003)

Data are presented as covariates-adjusted mean (SE) or percentage (SE). Adjusted means and proportions were calculated by using multivariable linear regression and logistic regression models with adjustment for age, education, alcohol and tobacco use, physical activity, alcohol drinking, smoking and survey sites. All P-values for trend across subgroups are <0.001. All P-values for gender difference are <0.001.

Table 3 shows gender-specific linear regression coefficients of spicy food eating for BMI, BF%, WC, and WHtR among regular spicy food consumers, adjusting for covariates as mentioned in Table 2. In model 1, both frequency and strength of spicy food eating showed significant and positive associations with BMI, BF%, WC, and WHtR except the associations of frequency with WC and WHtR in females and the strength-BF% association in males. In model 2, the years of eating spicy food-to-age ratio was significantly and positively associated with BMI, BF%, and WHtR in females and BF% in males. The inclusion of the years of eating spicy food-to-age ratio did not considerably change the association parameters of adiposity measures with frequency and strength of spicy food eating in both genders. No significant interactions were found between frequency, strength, and the years of eating spicy food-to-age ratio (data not shown). In the sensitivity analyses, the regression models in Table 3 were repeated in nonsmokers and nondrinkers. Only the association parameter for BMI was significant in males due to the small number of male subjects in this subgroup; however, the association patterns were substantially similar in females. Overall, the regression coefficients in the sensitivity analyses had the same directions as the original coefficients in Table 3 and were slightly greater, which justified the controlling for alcohol drinking and smoking as covariates in the original regression models.

**Figure 1:**
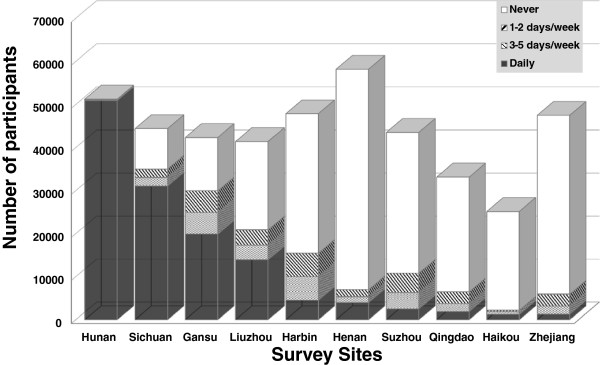
**Frequency of spicy food eating of participants in 10 survey sites of the CKB study.**

Figure 1 presents the number of participants of frequency of spicy food eating by survey sites. The survey site in Hunan province had the highest number of daily spicy food consumers (99.4%, n = 50,192); the survey site in Zhejiang province had the lowest number of daily eaters of spicy food (2.7%, n = 1,276).

**Figure 2:**
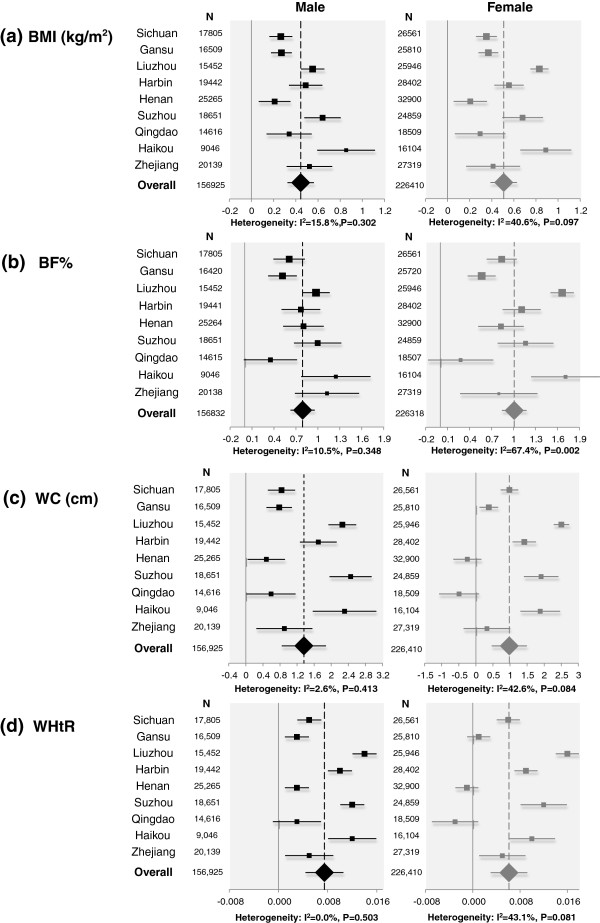
**Site-specific and overall effect of daily spicy food eating on BMI (a), BF% (b), WC (c) and WHtR (d) by gender groups.** Each closed square represents the point estimate of the regression coefficient, and the horizontal bar represents its 95% confidence interval (CI), which was estimated by using multiple linear regression models. Adjustment was made for age, study sites, education, alcohol and tobacco use, physical activity, alcohol drinking, smoking and survey site. The size of the square is proportional to the weight calculated by using the DerSimonian and Laird method. The overall point estimates, calculated in random effect models, are represented by dotted lines and closed diamonds, and the horizontal bar represents its 95% CI. BMI, body mass index; BF%, percentage body fat; WC, waist circumference; WHtR, WC-to-height ratio.

Since the prevalence of daily spicy food eating varied dramatically by survey sites, the overall effect of daily spicy food eating on adiposity measures also can be calculated by using meta-analysis. Figure 2 illustrates survey site-specific and overall effect of daily spicy food eating on BMI, BF%, WC and WHtR by gender groups. Hunan was excluded from the meta-analysis due to little variation in the frequency of daily eating (99.4%). By using the fixed effects model, all P-values for heterogeneity were <0.001; therefore, a random effects model was performed. Compared with non-consumers of spicy food, the pooled regression coefficients of daily spicy food eating of the nine sites were significant for BMI, BF%, WC and WHtR (0.44, 0.79, 1.36 and 0.007, respectively, in males; 0.51, 1.01, 0.98 and 0.006, respectively, in females).

**Figure 3:**
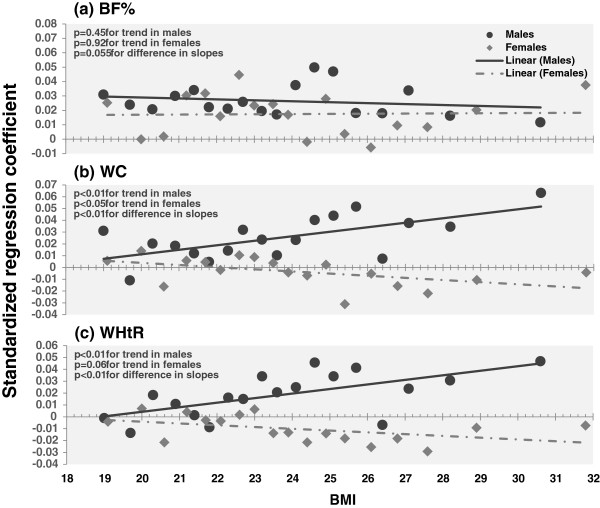
**Standardized regression coefficients of daily spicy food eating for adiposity variables by 18 BMI groups in males and females.** Absolute values of standardized regression coefficients ≥ 0.0338 in males and ≥0.0280 in females were significant (p < 0.05) for **a**; ≥ 0.0311 in males and ≥0.0311 in females for **b**; ≥ 0.0306 in males and ≥0.0216 in females for **c**. BMI, body mass index; BF%, percentage body fat; WC, waist circumference; WHtR, WC-to-height ratio.

In order to assess the association strengths of spicy food eating with BF%, WC, and WHtR at different BMI levels, males and females were stratified into 18 subgroups with equal numbers of subjects in each subgroup according to BMI levels. Figure 3 depicts standardized regression coefficients of daily spicy food eating for BF% (a), WC (b), and WHtR (c) by 18 BMI groups among males and females, adjusted for age, education, physical activity, alcohol drinking and smoking, and survey sites. Significant increasing trends in the associations between central obesity measures (WC and WHtR) and daily spicy food eating with increasing BMI were noted in males, but inverse trends were seen in females. No significant trends in BF% were observed in males and females.

**Table 3:** **Regression coefficients (SE) of spicy food eating associated with adiposity measures by gender groups among regular spicy food consumers** ^**a**^

	BMI		BF%		WC		WHtR	
	Males	Females	Males	Females	Males	Females	Males	Females
Model 1^b^								
n	78,858	105,988	78,808	105,931	78,858	105,988	78,858	105,988
Frequency of spicy food eating	0.078^**^	0.104^**^	0.207^**^	0.222^**^	0.192^**^	0.058	0.0010^**^	0.0003
	(0.017)	(0.016)	(0.034)	(0.033)	(0.052)	(0.045)	(0.0003)	(0.0003)
Strength of spicy food eating	0.068^**^	0.201^**^	0.051	0.334^**^	0.292^**^	0.560^**^	0.0018^**^	0.0035^**^
	(0.017)	(0.016)	(0.034)	(0.033)	(0.053)	(0.044)	(0.0003)	(0.0003)
Model 2^b^								
n	78,853	105,982	78,804	105,925	78,853	105,982	78,853	105,982
Frequency of spicy food eating	0.076^**^	0.096^**^	0.201^**^	0.207^**^	0.186^**^	0.050	0.0010^**^	0.0002
	(0.017)	(0.016)	(0.034)	(0.034)	(0.053)	(0.045)	(0.0003)	(0.0003)
Strength of spicy food eating	0.065^**^	0.193^**^	0.042	0.317^**^	0.282^**^	0.551^**^	0.0018^**^	0.0034^**^
	(0.018)	(0.016)	(0.035)	(0.034)	(0.054)	(0.045)	(0.0003)	(0.0003)
Years of eating spicy food-to-age ratio	0.011	0.023^**^	0.028^*^	0.046^**^	0.029	0.024	0.0001	0.0003^*^
	(0.007)	(0.006)	(0.013)	(0.013)	(0.021)	(0.018)	(0.0001)	(0.0001)

*p < 0.05, **p < 0.01.

^a^participants who ate spicy food more than once a week.

^b^Age, education, alcohol and tobacco use, physical activity, alcohol drinking, smoking and survey sites were included in models for adjustment.

## Discussion

Previous studies have shown a beneficial effect of spicy food consumption on weight management in intervention studies with a small sample size from Western countries [2, 6–8, 19], data are scant on the impact of spicy food on obesity in Asian populations. The CKB study, one of the largest cohort studies in the world, provides a great opportunity to bridge this gap. Despite the beneficial effect of spicy foods on body weight found in previous intervention studies [7, 8, 19, 25], a positive association of spicy food intake with BMI, BF%, WC, and WHtR was found in the current observational study. One potential explanation can be derived from differences between Eastern and Western dietary patterns. Unlike meat-based diet as typical Western staple foods, the plain and tasteless rice- or flour-made foods are staple foods for Chinese people. In light of the improvement in food flavor, taste, color, and smell, hedonic spicy food has been considered to increase palatability of meals and also been used against loss of appetite [2]. Thus, spicy food may increase carbohydrates intake of in Chinese population. In Chinese cuisines, spicy food are more meat-based rather than vegetable-based [26], with heavy salt and/or oil use for flavor or preservation, such as in hot pot, pickles, typically in Sichuan and Hunan regions. In this regard, excessive fat intake with spicy foods may increase the risk of obesity. Additionally, hot pepper consumption proved to increase desire for sweet foods [8] which helps cool the heat and relieve the pain of the tongue. Therefore, additional intake of sweet foods and beverages along with or after spicy food eating may also contribute to the positive association of spicy foods intake with obesity.

It is known that perception or liking of taste, olfaction, and somatosensory sensations varies with environmental exposures, genetics, aging, and their complex interactions [27]. The marginal level of weight-loss effects (an estimated increase of 50 kcal/day energy expenditure) [28, 29] of spicy food can be easily biased. Previous studies have shown that a former exposure to spicy food may attenuate the weight-loss effects, due to an induced adaption to thermogenic, appetitive, and other weight-loss effects [6, 8, 19, 25]. Therefore, the slimming effects of spicy food consumption on BMI, BF%, WC, and WHtR cannot be found in the current study, since participants ate spicy food at different frequencies as a lifetime eating habit. Moreover, Inoue-Choi et al. [30] found polymorphism in COMT (catecholamine O-methyl transferase) affected slimming effects of green tea, which suggests that sensitivity to weight-loss effects of spicy food might be modulated by genetic variations as well, for instance, TRPV1 (the transient receptor potential cation channel subfamily V member 1) and TRPA1 (the transient receptor potential cation channel subfamily A member 1) [31, 32].

Although prediction values of risk factors for cardiovascular disease were similar in Chinese and U.S. adults [33]. Asian populations have a lower BMI, but a greater prevalence of central obesity than Caucasian populations [9, 23, 34–36]. Daily spicy food eating was positively associated with WC and WHtR in males; the higher BMI was, the greater the effects became. However, in females, the trend in the effect of spicy food intake on WC and WHtR decreased with increasing BMI in stratified analyses, and spicy food intake was negatively associated with WC and WHtR in most subgroups with a BMI greater than 24. Gender difference in food choice might represent one of the possible reasons for the inverse association of spicy food with central obesity in females. As females are reported to eat more fruit and vegetables but less high-fat foods than males [37, 38], vegetable-based spicy foods could be good for health; for example, chili pepper is rich in vitamin C, vitamin A and β-carotenoids. Moreover, a Korean study showed that a 12 weeks’ introduction of spicy sauce (Kochujang) to daily diet resulted in visceral fat loss and better lipid profiles, but without weight change [39].

The current observational study demonstrated a real-world spicy food intake and its association with general obesity and central obesity, rather than an intervention study or a clinical trial with relatively short period effects with a standardized meal of capsaicin-contained foods (e.g. red pepper) or supplement [25]. A cohort of nearly half a million participants with demographic, cultural, and socioeconomic diversity not only gave us the descriptive data on eating patterns of spicy food, but also provided sufficient power to control confounding and a large sample for stratified analyses. Compared with the findings from previous intervention studies, the observation on the positive association between spicy food eating and obesity in the current Chinese population-based study provided information on the difference in dietary patterns, related health behaviors or even gene-environment interactions between Western and Chinese populations.

This population-based epidemiologic study has certain limitations. First, detailed analyses such as interaction effects, although important, have not been examined due to the limited space in one manuscript for publication. Second, the pathophysiological mechanisms underlying the effect of spicy food eating on obesity cannot be investigated in the current study because the baseline survey data are observational in nature. Finally, the CKB study is not a nutritional survey, and detailed information on dietary patterns is not available. Therefore, dietary factors, which may be important, were not included in the current analyses.

## Conclusion

The current study describes spicy food consumption in the Chinese adult population and demonstrates that spicy food intake is positively associated with BMI, BF%, WC, and WHtR regardless of gender. With adjustment for BMI in stratified analysis, spicy food eating is associated with central obesity in males, but this effect is weaker in females. The findings of this study indicate that daily consumption of spicy food may be a risk factor for overweight and obesity in Chinese adult population. Additional research is needed to confirm this finding in terms of obesity-related dietary risk factors, clustering of unhealthy lifestyles, and genetic variance.
